# Short Communication: Correlation of Thermographic Ocular and Auricular Temperatures with Rectal Temperature in Anesthetized Dogs

**DOI:** 10.1155/2023/9939580

**Published:** 2023-10-19

**Authors:** Ayse Basak Kapcak, Elif Dogan

**Affiliations:** Kastamonu University, Veterinary Faculty, Department of Surgery, Kastamonu, Turkey

## Abstract

**Objective:**

The aim of this study was to determine the correlation of both ocular and auricular thermographic temperatures with rectal temperatures and to determine the advantage of infrared thermography in clinical practice due to its ease of measurement. *Animals*. This study was designed as a pilot study and conducted on 6 mongrel male dogs during routine castration surgeries at the Kastamonu Municipality Temporary Animal Care Center.

**Methods:**

Rectal temperatures and ocular-auricular thermographic images were taken from 6 dogs anesthetized (xylazine HCl 1 mg/kg and ketamine HCl 15 mg/kg) during routine neutering surgeries. Data were analyzed by Bland–Altman for correlation between rectal-ocular and rectal-auricular temperatures.

**Results:**

Rectal temperatures were significantly higher than orbital and auricular temperatures. In the correlation test, no significant difference and correlation were found between the measurements.

**Conclusion:**

As a result of the study, it was determined that the use of thermography was more advantageous than the waiting times of the digital thermometer used to record rectal temperatures. In addition, the noninvasive nature of thermography increased its acceptability in all dogs. The findings from this pilot study were considered to be at a level that could provide a basis for future studies.

## 1. Introduction

With the induction of general anesthesia, body temperature is redistributed from the core to the environment. It is lost through convection and evaporation during anesthesia [1]. A decrease of 1–1.5°C in temperature during the first hour of anesthesia and 2.5–3°C in the following hours can be observed [2]. Hypothermia cases seen in 83% of dogs under general anesthesia [3] increase the risk of postoperative infection and lead to cardiovascular complications and longer recovery [4]. The rectal temperature used for monitoring body temperature is approximately 0.4°C lower than the core temperature in dogs [5]. The disadvantages of rectal measurement include the presence of air, stool, or masses in the intestines and the measurement may change [6], discomfort in rectum, anus or pelvic diseases [7], and changes in blood pressure and pulse as a result of the defensive behaviors of patients [8]. For these reasons, it is being investigated whether measuring different surface temperatures with infrared thermography (IRT) is an alternative [9]. This method [10], which allows the detection of real-time changes in body surface temperatures, is considered advantageous in farm animals and pets as it is a nonstress-inducing, noncontact technique [11]. In small animal medicine, changes in blood circulation can cause shifts in surface temperature, indicating inflammation or disease. Therefore, thermographic examination in dogs has the potential to be a diagnostic tool [12]. Significant correlations have been reported between the rectal temperature and surface temperature taken with IRT in dogs [9]. However, there is little information on thermal windows other than extremities from thermography studies in dogs [13].

Since the eardrum has the potential to reflect core body temperature, temperature measurements are taken from around the ear [14]. Studies in dogs have reported a strong correlation between rectal temperature and auricular IRT measurements [15, 16]. The auricle structures of the dogs used in the studies can change the temperature readings. Therefore, whether it is an alternative to the rectal temperature should be evaluated with different dog breeds [6]. There have also been studies examining the correlation of ocular temperatures with auricular measurements [17]. The first ocular temperature study based on measurement of ocular surface temperature changes due to tear film transmission [18] was made in 1968 from different parts of the eyeball [19, 20]. Studies have shown that the ocular temperature taken with IRT is a potential tool, especially in animal welfare. The medial palpebral border and lacrimal cartilage, a small area with rich capillary beds innervated by the sympathetic nervous system in the eye tissue, are suitable for measurement [21]. Thermographic ocular temperature measurements can be made faster than the measurements taken from the rectum with a digital thermometer. The noninvasiveness of this method used ensures that regional temperatures can be obtained easily in all species [22].

Studies involving the body temperature of dogs have evaluated temperature differences in limited superficial areas in response to certain clinical conditions [23]. In this pilot study, it was aimed to thermographic examination of thermal windows that can be used easily in the clinic and an alternative to the rectal route, under general anesthesia in dogs. In the measurements taken, it was aimed to use areas with easy access and high tolerance for both clinicians and animal owners.

## 2. Materials and Methods

### 2.1. Animals and Measurements

The presented study was designed as a pilot study and was carried out in 6 crossbred, 2-3 years old, male dogs that were anesthetized by the veterinarian during the routine castration operations in the Kastamonu Municipality Temporary Animal Care Center. The dogs were confirmed to be healthy by the veterinarian examination. Before anesthesia, the dogs were taken to an empty room and allowed to acclimate to room temperature for 15 minutes [24]. At the end of the period, the animals were administered general anesthesia with xylazine HCl (1 mg/kg, IM, Control, Turkey) and ketamine HCl (15 mg/kg IM, Keta-Control, Turkey). The rectal temperature was measured with a digital thermometer (Kerbl, Germany), and orbital and auricular temperatures were measured with a thermography device (FLIR Systems, Inc., Sweden) 15 minutes after induction of anesthesia.

The emissivity value was set to 0.97 as stated in literature [25]. The distance between the dog and the camera was set to an average of 0.50 m to capture thermal images from the specified areas [26]. Room temperature was maintained at 21°C for all procedures. The same thermography device was used for all imaging to reduce user error variability.

### 2.2. Statistical Analysis

All results were expressed as the mean ± SD. The Brown–Forsythe test and Bartlett's test were used to determine whether the data were normally distributed. A paired Student's *t*-test was used to assess statistical differences between the left-right eye and the left-right ear. No statistical difference was observed between symmetrical organs, so mean values were used for subsequent statistical analysis. The Bland–Altman test was used to evaluate statistical differences between rectal, ocular, and auricular temperatures.

## 3. Results

**Figure 1:**
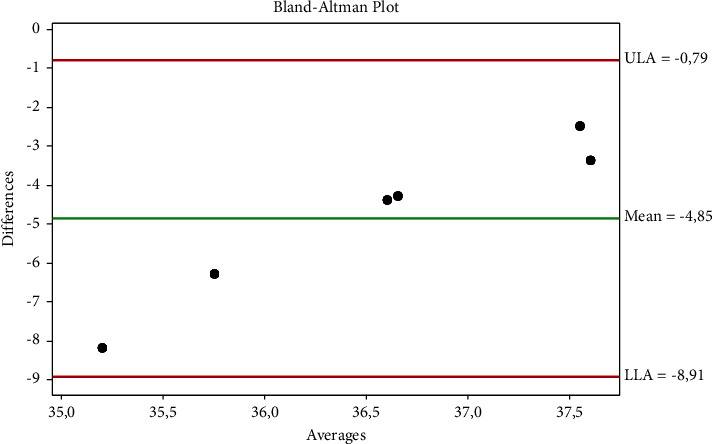
The agreement between auricular-rectal temperatures.

**Figure 2:**
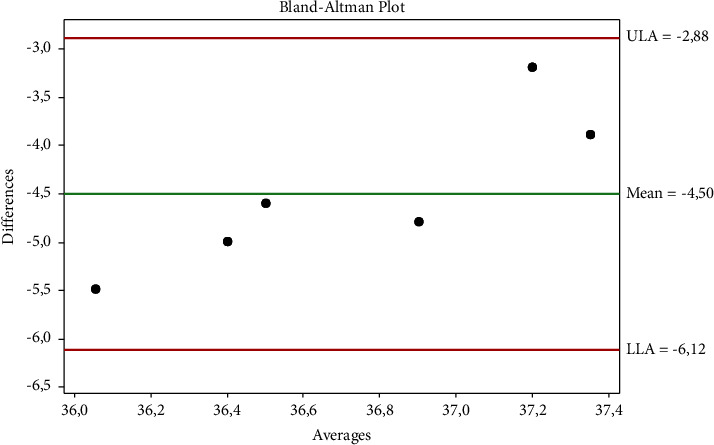
The agreement between ocular-rectal temperatures.

**Figure 3:**
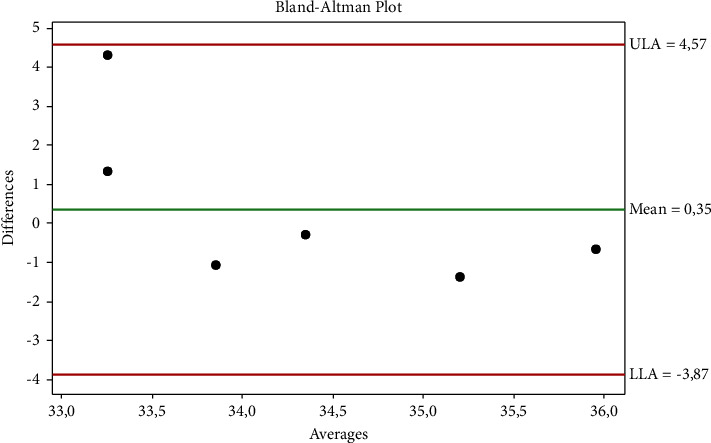
The agreement between ocular-auricular temperatures.

**Figure 4:**
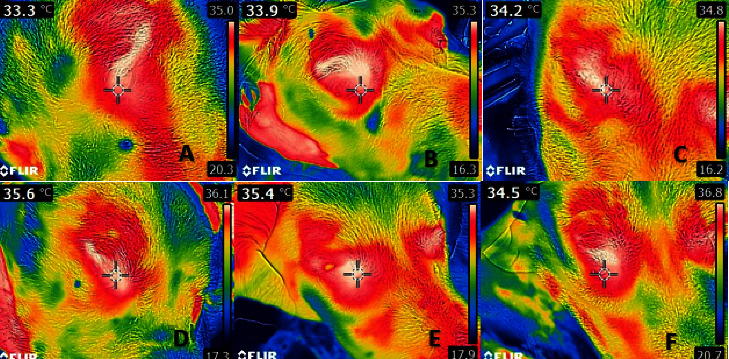
Thermographic images from dogs: (A) 1st dog's ocular thermography, (B) 2nd dog's ocular thermography, (C) 3rd dog's ocular thermography, (D) 4th dog's ocular thermography, (E) 5th dog's ocular thermography, and (F) 6th dog's ocular thermography.

**Figure 5:**
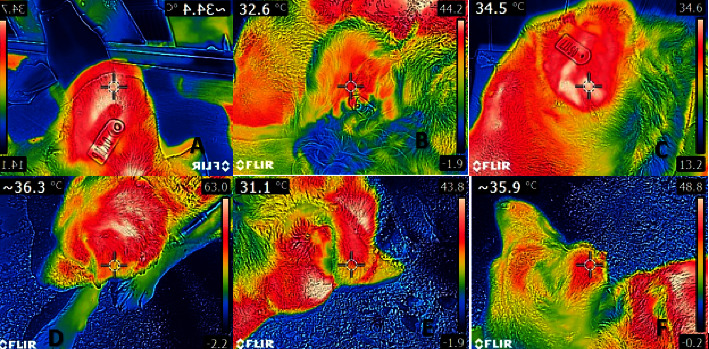
Thermographic images from dogs: (A) 1st dog's auricular thermography, (B) 2nd dog's auricular thermography, (C) 3rd dog's auricular thermography, (D) 4th dog's auricular thermography, (E) 5th dog's auricular thermography, and (F) 6th dog's auricular thermography.

The Brown–Forsythe test and Bartlett's test showed that the data were normally distributed (*p* ˃ 0.005).  Table 1 shows that ocular-rectal, auricular-rectal, and ocular-auricular temperatures were not in agreement. The agreement between auricular-rectal temperatures is shown in Figure 1, the agreement between ocular-rectal temperatures is shown in Figure 2, and the agreement between ocular-auricular temperatures is shown graphically in Figure 3. Thermographic measurements taken from dogs are shown in Figures 4 and 5.

## 4. Discussion

This study aimed to noninvasively assess body temperature under xylazine-ketamine general anesthesia in dogs. Many anesthetic agents cause vasodilation with a dose-dependent effect on peripheral vascular tone, leading to peripheral dissipation of heat [27]. In this study, which was designed based on this known effect of general anesthetics, only xylazine-ketamine anesthesia was applied. Further studies are planned to compare the changes that occur with the use of other anesthetics. It has been reported that up to 89% of anesthetized dogs have a decrease in body temperature during recovery [28] and that this decrease occurs in the first 15 minutes of anesthesia [29]. Therefore, in our study, temperature measurements were made 15 minutes after induction of anesthesia. Our primary focus was to evaluate the correlation between the rectal temperature, which is considered the gold standard, and the thermal windows. Our other focus was to investigate an alternative noncontact way for body temperature measurements that cannot be taken rectally. Surface temperatures from thermal windows are measured by infrared thermography, and measurement locations are the main factor affecting accuracy [15]. The thermal window of the eye, especially the lacrimal cartilage, has rich capillary beds that respond to blood flow changes in humans [30], cattle [31], and dogs [32]. This area also has the advantage that it is not affected by body size [16]. In addition, the fact that the eardrum also has the potential to reflect body temperature and that studies have been conducted by taking measurements from around the ear [14] led to the selection of the ear region as the second thermal window along with the ocular thermal window in our study. In addition to studies reporting that both ocular and auricular temperatures are significantly correlated with the rectal temperature [15, 33], there is also research that suggests that measurements do not reflect actual temperature and are influenced by environmental factors [34]. This pilot study was planned due to few studies on thermal windows in veterinary medicine [13]. Since there was no statistical difference between symmetrical organs in our study, temperature averages were taken into consideration. This finding is consistent with the results of the study conducted by Giannetto et al. [35] in cats. It has also been reported that the right or left ear can be used safely due to the lack of anatomical differences for auricular measurements [16]. The temperatures recorded from the ocular and auricular thermal windows were significantly lower in our study, as reported in previous studies [33, 36]. The other finding was that although ocular and auricular temperatures were significant compared to the rectal temperature, there were no significant difference and correlation between them. However, Zanghi [15] concluded that in resting dogs, auricular measurements are more accurate than ocular measurements. At this point, we can say that xylazine-ketamine anesthesia did not have a variable effect on ocular and auricular measurements. However, we think that this difference between resting and under anesthesia should be investigated in detail.

Wiedemann et al. [37] reported a moderate correlation between ocular and rectal temperatures in dogs and that this region can be used as an alternative. Kunkle et al. said the same about the ocular temperature [9]. We could not detect a correlation between the measurements in our study [38]. We think this is the result of the small number of animals studied as it was a pilot study. There are several limitations in this study. The limitations of the study are the small number of animals, only xylazine-ketamine anesthesia, and lack of measurement repetitions.

## 5. Conclusion

In conclusion, the present study was conducted in the small number of animals as it was a pilot study, and measurements were taken from dogs that were already under anesthetic. In order for infrared thermography to be used in the assessment of body temperature, the anatomical region to be compared with the rectal temperature must be studied in detail. It is necessary to determine the correlations of surface temperatures taken from different anatomical regions with rectal temperatures and within themselves. It is possible to reach more accurate results by evaluating racial characteristics in thermographs taken from anatomical regions. In our study, measurements were taken only in anesthetized dogs, but to determine the effect of anesthesia on these temperatures, we recommend the use of different anesthetic agents, taking data in the pre- and post-anesthesia period, and also making measurements at different times during deep anesthesia. The use of the thermal camera allowed rapid acquisition of ocular and auricular temperatures compared to the dwell times of the digital thermometer used to record the rectal temperature. In addition, thermography is noninvasive, which increases its acceptability in all animal species.

## Figures and Tables

**Table 1:** Statistical evaluation of the temperature measurements.

Temperature (°C)	Rectal	Ocular	Auricular	
Mean ± SD (95% CI)	38.98 ± 0.24	34.48 ± 0.88	34.13 ± 1.97	
	(38.72–39.24)	(33.56–35.41)	(32.06–36.21)	

	d ± 1.96 s	Sd	Compliance limits	

Ocular vs. rectal	−4.50	0.82	−2.88	−6.12
Auricular vs. rectal	−4.85	2.07	−0.79	−8.91
Ocular vs. auricular	0.35	2.15	4.57	−3.87

Sd: standard deviation.

## Data Availability

The data that support the findings of this study can be obtained from the corresponding author upon request. The statistical study of the data is given in the additional file.
